# Combined focal myoclonus and dystonia secondary to a cerebellar hemorrhage: a case report

**DOI:** 10.1186/s12883-016-0745-6

**Published:** 2016-11-17

**Authors:** Guangxun Shen, Guangxian Nan, Chae-Won Shin, Hyeyoung Park, Kwee-Yum Lee, Beomseok Jeon

**Affiliations:** 1 Department of Neurology, China-Japan Union Hospital of Jilin University, Changchun, China; 2 Department of Neurology, SNU-SMG Boramae Medical Center, Seoul, Republic of Korea; 3 Department of Neurology, Seoul National University College of Medicine, Seoul National University Hospital, 101 Daehak-ro, Jongno-gu, Seoul, 110-774 Republic of Korea; 4 School of Medicine, University of Queensland, Brisbane, QLD 4072 Australia

**Keywords:** Myoclonus, Dystonia, Cerebellar lesion, Magnetic resonance imaging, Electromyography

## Abstract

**Background:**

Myoclonus is a clinical sign characterized by sudden, brief jerky, shock-like involuntary movements of a muscle or group of muscles. Dystonia is defined as a syndrome of sustained muscle contractions, frequently causing twisting and repetitive movements or abnormal postures. Cases of myoclonus or dystonia secondary to a structural lesion in the cerebellum have been reported. However, there has never been a reported case of combined myoclonus and dystonia secondary to a cerebellar lesion.

**Case presentation:**

Herein, we report a 22-year-old female patient with sudden-onset myoclonic jerks, dystonic posture and mild ataxia in the right upper extremity. At age 19, she experienced sudden headache with vomiting. The neurological examination showed ataxia, myoclonus and dystonia in the right upper extremity. Brain images demonstrated a hemorrhage in the right cerebellar hemisphere secondary to a cavernous malformation. After resection of the hemorrhagic mass, headache with vomiting disappeared and ataxia improved, but myoclonus and dystonia persisted.

**Conclusions:**

It is the first report of combined focal myoclonus and dystonia secondary to a cerebellar lesion.

**Electronic supplementary material:**

The online version of this article (doi:10.1186/s12883-016-0745-6) contains supplementary material, which is available to authorized users.

## Background

Myoclonus is a clinical sign characterized by sudden, brief jerky and shock-like involuntary movements of a muscle or group of muscles. The main anatomical origins of the myoclonic jerks are known to be cortical, subcortical, spinal, and peripheral. Although rare, several cases of myoclonus secondary to a structural lesion in the cerebellum have been reported. Therefore, it has been speculated that cerebellum might be one of the possible generators of myoclonus [1]. Progressive myoclonus was reported in a young child with a ganglioglioma in the region of the deep cerebellar white matter and nuclei [2].

Dystonia is defined as a syndrome of sustained muscle contractions, frequently resulting in twisting and repetitive movements or abnormal postures. Although most common structural lesions responsible for dystonia are in basal ganglia, cerebellum is also considered to be important in dystonia [3, 4]. Dystonia secondary to a cerebellar lesion is rare, but several cases have been reported in the literature. For example, focal limb dystonia was demonstrated in a patient with a cerebellar mass [5]. Herein, we describe an unusual case of sudden-onset combined focal myoclonus and dystonia secondary to a cerebellar lesion.

## Case presentation

**Fig. 1:**
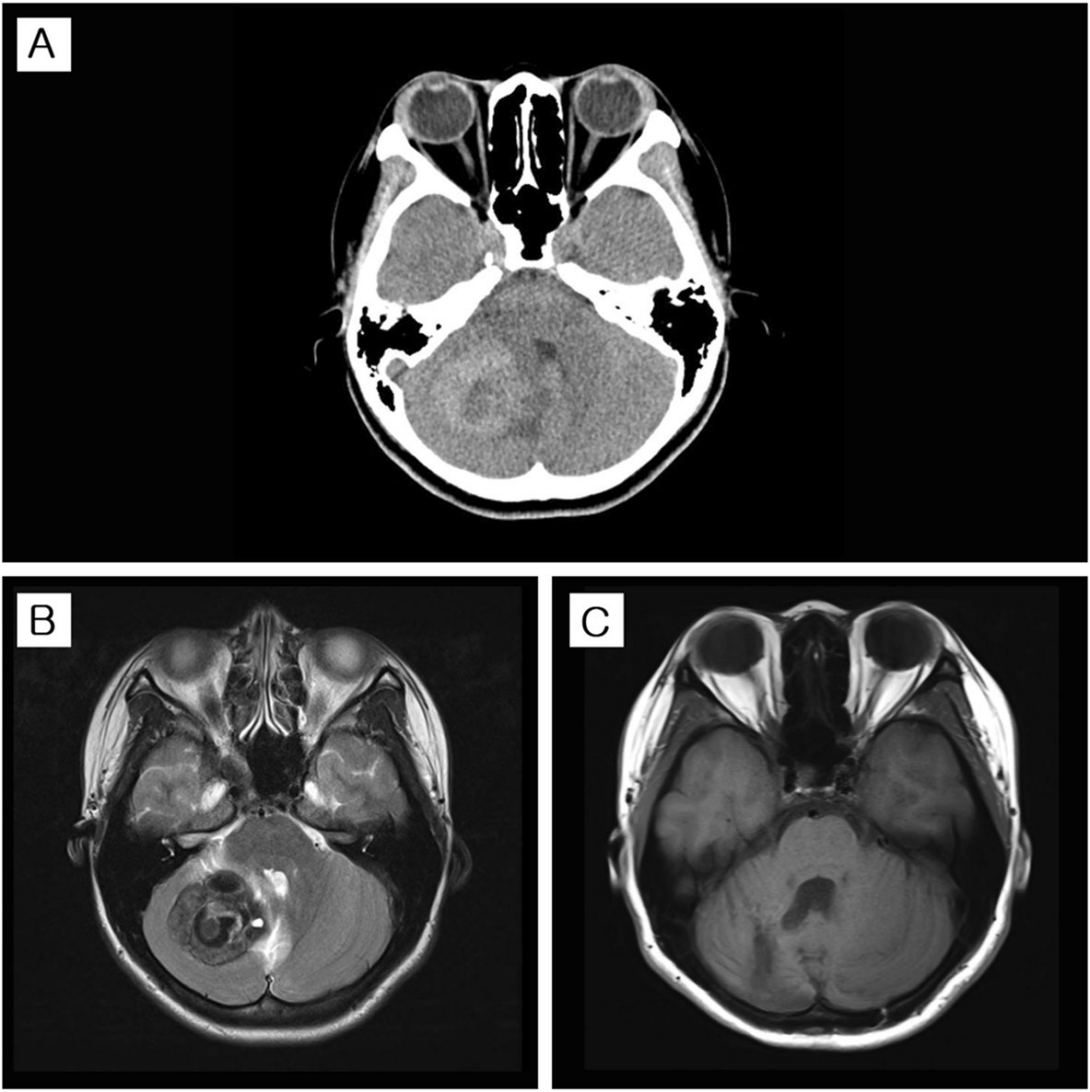
Brain images. Axial CT scan at the onset of the symptoms demonstrated a hemorrhagic mass in the right cerebellar hemisphere and the fourth ventricle compressed by the mass (**a**). Axial T2-weighted MRI demonstrated heterogeneous low signal intensity of the mass (44 × 37 mm) with high signal intensity in the periphery (**b**). Axial T1-weighted MRI at 15-month follow-up after resection demonstrated a post-operative malacic lesion in the right cerebellar hemisphere (**c**)

A 22-year-old right-handed woman was referred for management of jerky movements and abnormal posturing of the right hand that had developed since acute hemorrhage in the right cerebellar hemisphere secondary to a cavernous malformation. At age 19, she developed an episode of severe headache with vomiting, and poor coordination. Examination revealed ataxia in her right upper extremity along with jerks and abnormal posturing of her right hand and fingers. Brain computed tomography (CT) and magnetic resonance imaging (MRI) showed a hemorrhage in the right cerebellar hemisphere compressing the fourth ventricle (Fig. 1a and b). No structure lesions were found in the supratentorial region. Resection of the mass and pathological examination confirmed the diagnosis of cavernous malformation. Her headache improved after surgery. However, mild ataxia in the right arm, abnormal posturing of the hand and spontaneous and sudden jerks of the right fingers persisted. Furthermore, the jerks were unable to be suppressed voluntarily but disappeared during sleep. Alcohol intake did not seem to affect those abnormal movements, but anxiety and stress worsened the symptoms.

Neurological examination at our clinic showed mild dysmetria in the right upper extremity on the finger-to-nose test. The right hand showed occasional brisk jerks at rest which became more evident on maintaining posture, but were not provoked by touch. Abnormal posturing of the fingers was noted, which was accentuated while outstretching. An abnormally tight grip was observed while writing with a pencil. (See Additional file 1, a video footage of the patient showing these features in more detail). The patient presented symmetrical deep tendon reflex and intact proprioceptive sensation. No long-tract signs were found. There were no changes in the severity of these involuntary movements during a 6-month trial of piracetam, biperiden, baclofen and clonazepam.


Additional file 1: A 22-year-old woman with focal myoclonus and dystonia in the right upper extremity. No myoclonus is noted at rest or triggered by external stimuli. Postural myoclonus of the right hand is prominent and her right fingers show dystonic posturing when she outstretches the arms and rotates the wrist. The abnormal posturing of hand also interferes with a writing task. The patient shows normal gait.


**Fig. 2:**
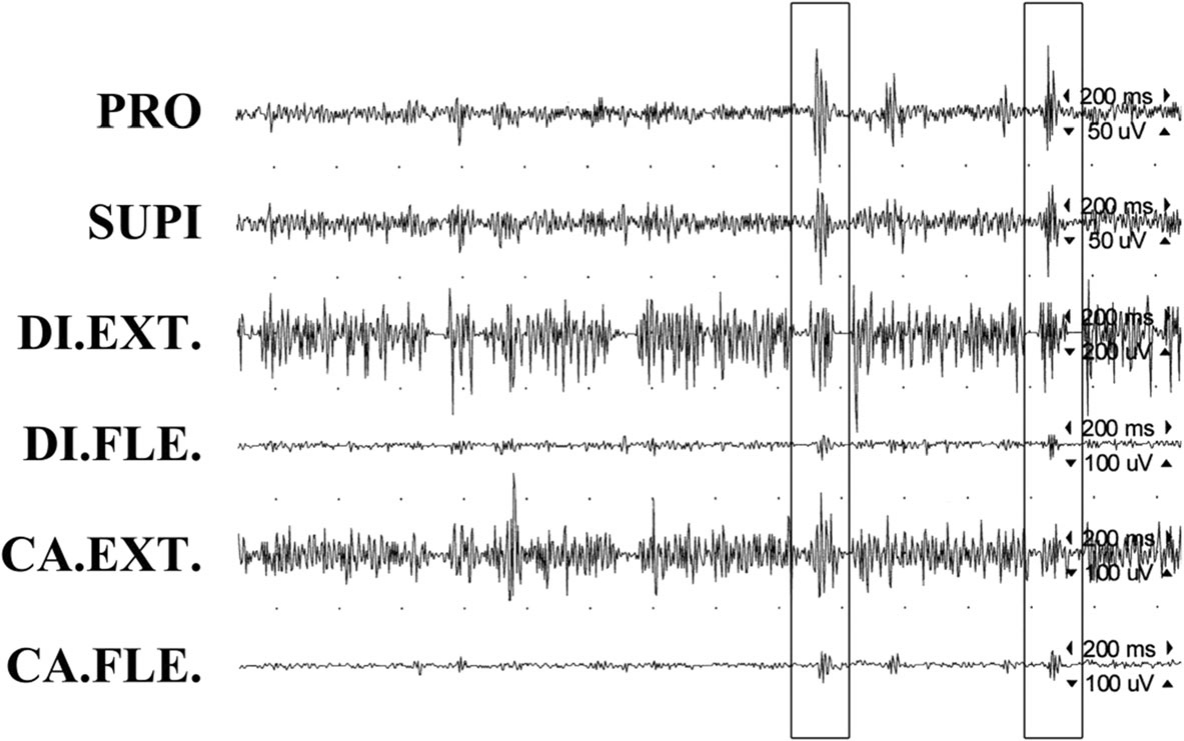
Surface EMG. EMG recordings of the right upper extremity while maintaining a posture demonstrate two consecutive jerks (93–97 ms), showing synchronous bursts in agonist and antagonist muscles. PRO: pronator teres muscle; SUPI: supinator muscle; DI. EXT: extensor digitorum communis muscle; DI. FLE: flexor digitorum profundus muscle; CA. EXT: extensor carpi ulnaris muscle; CA. FLE: flexor carpi radialis muscle

A 15-month follow-up MRI demonstrated a post-operative malacic lesion in the right cerebellar hemisphere (Fig. 1c). Surface electromyography (EMG) recordings of the right forearm while keeping outstretched illustrated the typical features of myoclonus with synchronous bursts (93–97 ms) of EMG activities in agonist and antagonist muscles (Fig. 2).

## Conclusion

Our patient presented with sudden-onset shock-like jerks in her right fingers, superimposed on more sustained abnormality of posture in the right hand with mild ataxia in the right arm. Surface EMG recordings confirmed the jerks as myoclonus, which were synchronous and short-lasting activities in agonist and antagonist muscles. Dystonic posturing of the right hand was evident in clinical examination (Additional file 1). It is most likely that the cerebellar lesion is responsible for both myoclonus and dystonia in our case based on the following reasons: 1) The onset of the myoclonus and dystonia coincided with onset of ataxia; 2) The lesion of the right cerebellar hemisphere was demonstrated on the brain CT and MRI; and 3) Involuntary movements were ipsilateral to the cerebellar lesion.

Cerebellum as a structure in cerebro-cerebellar loops, participates in motor control by means of sensorimotor integration - the process whereby sensory input is integrated by the central nervous system and used for assisting motor program execution [6, 7].

Even though there have been reports of dystonia in cerebellar lesion, the pathophysiology is not entirely clear. However, there are suggestions that abnormal cerebellar learning and functional interactions between cerebellar and basal ganglia circuits may play a role [8, 9].

Role of cerebellum in myoclonus is also not clear. However, there are several reported cases of cortical myoclonus in patients with cerebellar pathology, which are documented by neurophysiology and pathology [10]. This paradoxical absence of cortical pathology and cortical myoclonus in patients with cerebellar pathology has promoted the authors to suggest that enhanced excitability of the sensorimotor cortex may arise as a distant effect of cerebellar pathology [10]. This has further been explored in the review by Ganos et al. [11].

Our case is unique in that the hemorrhage in the right cerebellar hemisphere and subsequent surgical operation result in myoclonus and dystonia at the same time, and it further supports the role of cerebellum in the generation of myoclonus and dystonia.

In conclusion, the findings in our patient support that movement disorders resulting from lesions of the cerebellum may include myoclonus and dystonia.

## Abbreviations

CT: Computed tomography
EMG: Electromyography
MRI: Magnetic resonance imaging
